# Profile of phenolic compounds and antioxidant activity of organically and conventionally grown black-grain barley genotypes treated with biostimulant

**DOI:** 10.1371/journal.pone.0288428

**Published:** 2023-07-12

**Authors:** Rafał Nowak, Małgorzata Szczepanek, Joanna Kobus-Cisowska, Kinga Stuper-Szablewska, Marcin Dziedziński, Karolina Błaszczyk

**Affiliations:** 1 Department of Agronomics, Faculty of Agriculture and Biotechnology, Bydgoszcz University of Science and Technology, Bydgoszcz, Poland; 2 Department of Gastronomy Sciences and Functional Foods, Poznan University of Life Sciences, Poznan, Poland; 3 Department of Chemistry, Poznan University of Life Sciences, Poznan, Poland; Bangabandhu Sheikh Mujibur Rahman Agricultural University, BANGLADESH

## Abstract

Beneficial dietary and pro-health values, have resulted in the increasing consumption importance of barley. Therefore, genotypes and cultivation methods are sought that guarantee high functional value of grain. The aim of the study was to assess the content of phenolic acids, flavonoids, chlorophylls, anthocyanidins, phytomelanin and antioxidant activity of grain of three barley genotypes depending on agricultural technology. Two of them are primary genotypes with dark grain pigmentation *Hordeum vulgare* L. var *nigricans* and *H*. *vulgare* L. var. *rimpaui*, the third is a modern cultivar ’Soldo’ *H*. *vulgare* with yellow grain, which is the control sample. Evaluated the effect of foliar application of a amino-acids biostimulant on the functional properties of grain under the conditions of organically and conventionally cultivations. The results indicated a higher antioxidant activity and the concentration of phenolic acids, flavonoids and phytomelanin in the black-grain genotypes. Organic cultivation and application of amino acids had increased the content of phenolic compounds in grain. The antioxidant activity was correlated with the content of syringic acid, naringenin, quercetin, luteolin and phytomelanin. Organically cultivation and the foliar application of an amino acid biostimulant improved the functional properties of barley grain, in particular the original, black-grained genotypes.

## Introduction

The genus barley (*Hordeum* spp.) belonging to the tribe *Triticeae* includes more than 30 species found in temperate climate as well as dry and low-fertile regions of the world. The most economically important species of this genus is common barley (*Hordeum vulgare*). It is the fourth crop after wheat, rice and maize in terms of economic importance. It is used for fodder purposes, in the brewing industry and in human nutrition [1]. In recent years, the consumption importance of barley has increased due to its health-promoting properties. The high dietary values of barley grain result, among other things, from the content of bioactive substances with antioxidant properties, mainly phenolic compounds such as phenolic acids, flavonols or anthocyanins [2]. Antioxidants reduce oxidative stress by scavenging free radicals, thanks to which they have a protective effect on the body cells and reduce the risk of neurodegenerative diseases, e.g. Alzheimer’s and Parkinson’s and heart diseases. Antioxidants also protect the cells of organisms from the harmful effects of heavy metals by chelating them [3]. Antioxidants also have a protective effect on the plant itself, limiting the effects of abiotic and biotic stress [4]. For example, they reduce the infestation of barley by pathogenic fungi and can reduce the amount of mycotoxins produced by pathogens [5]. The concentration of antioxidants in the green parts of plants, as well as in the cereal grain itself, depends on environmental and genetic factors as well as cultivation technology [6]. Some primary or close to primary barley genotypes show high antioxidant potential, especially those with black-colored grains [7]. Barley grain can be yellow, purple, blue or black. The first three colors result from the content of flavonoids, while the black color is caused by phytomelanins which are formed from oxidized and polymerized phenolic compounds [8]. It can therefore be assumed that the black-colored seed of the primary barley genotypes: *Hordeum vulgare* L. var *nigricans* (Ser.) Körn and *H*. *vulgare* L. var. *rimpaui* Wittm. can be a valuable raw material for the production of functional food.

According to Baker et al. [9], breeding and development of high-quality barley, especially useful for cultivation in an organic farming system, is very important for expanding the markets and increasing the potential of barley cultivation. In the conditions of organic cultivation, there is a high exposure of plants to stress factors, which increases the production of bioactive compounds [10]. Many researchers have also proven the effect of the application of amino acid biostimulants on the increase in the content of phenolic compounds in plants, for example in soybean seeds [11, 12], pepper fruits [13] and lettuce leaves [14]. Phenolic compounds are formed in the phenylpropanoid pathway from amino acids [15]. However, the literature lacks studies on the effect of use of amino acid biostimulants on the content and composition of bioactive compounds, including phenolic compounds, in barley grain. There are also no studies on the impact of this factor on the quality characteristics of barley grain grown in organic and conventional farming systems.

Previous research on the genotypes of black-coloured barley has generally focused on the influence of genetic factors on the quality characteristics of the grain [8]. However, the impact of agrotechnical factors such as the management system and the application of biostimulants on the bioactive compound content of the grain was not considered. As suggested by the literature [7, 8, 16] it can be considered that these genotypes, due to their potentially high health value, may show great suitability for organic cultivation.

The aim of this study was to determine the differences in the profile of phenolic compounds and the antioxidant potential of barley between the primary black-grain genotypes of barley and the modern yellow-grain cultivar. The study was also to show whether the use of a biostimulant consisting of free amino acids would affect the content of phenolic compounds and the antioxidant potential of barley grain under the conditions of organic and conventional farming systems.

This research will expand the knowledge about the impact of the farming system and the application of amino acid biostimulant on the quality characteristics of barley grain. The response of the original *Hordeum vulgare* L. genotypes to agrotechnical factors will also be recognized, in the context of the quality characteristics of the grain.

## Material and methods

### Plant materials

**Fig 1:**
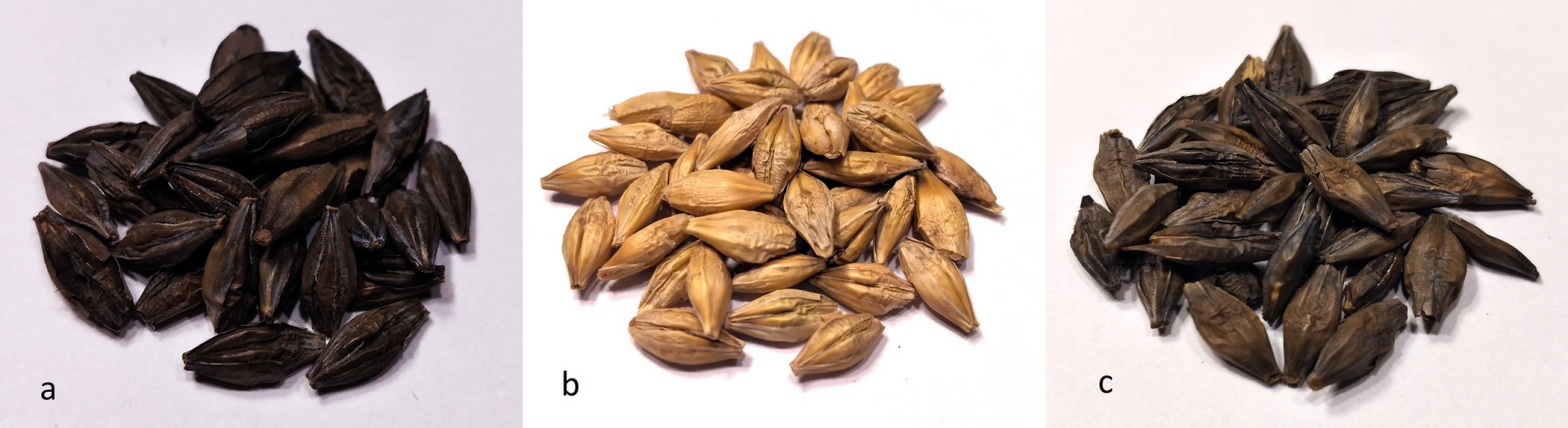
Grains of the barley genotypes evaluated in the research–(a) Hordeum vulgare L. var. nigricans (Ser.) Körn, (b) Hordeum vulgare ’Soldo’, (c) H. vulgare L. var. rimpaui Wittm.

The research material consisted of dark colored grain of two primary barley genotypes *Hordeum vulgare* L. var *nigricans* (Ser.) Körn and *H*. *vulgare* L. var. *rimpaui* Wittm and yellow grain of the modern cultivar ’Soldo’ of common barley *(H*. *vulgare* L.) (Fig 1). The first two genotypes are spring forms of common barley, belonging to the subspecies of two-row barley–ssp. *distichon*. They form hulled grains, dark brown to black in color, set on a loose and drooping spike (var. *nutans*). *Hordeum vulgare* L. var *nigricans* (Ser.) Körn forms the awned spike, while *H*. *vulgare* L. var. *rimpaui* Wittm has awns transformed into hoods—beardless barley (var. *trifurcatum* or *furcatum*). The cultivar ’Soldo’, treated in the study as the control for the two previous genotypes, is one of the very popular cultivars of spring, two-rowed barley in Europe. Its breeder is Saaten Union. It belongs to hulled cultivars, it is recommended for cultivation for consumption or fodder purposes. It is characterized by high and stable yields in conditions of high and medium level of agricultural technology. It is characterized by medium tillering and high thousand grain weight. It is a cultivar with high resistance to the most important diseases [17]. It shows also suitability for cultivation in an organic system.

### Field experiments

The grain of three tested barley genotypes subjected to analyses came from strict field experiments, established in a randomized sub-blocks design (split-plot) in 3 replicates, in which the response of barley to the foliar application of an amino acid biostimulant Naturamin WSP (Daymsa, Spain) was assessed. The main plots consisted of barley genotypes: i) *H*. *vulgare* L. var. *rimpaui* Wittm, ii) *H*. *v*. L. var. *nigricans* (Ser.) Körn, iii) ‘Soldo’ *H*. *vulgare*, and the subplots were biostimulant treatments: i) control without application, ii) a foliar application of amino acid biostimulant twice a season. The plot area in the conventional system was 24 m^2^ (16 m x 1.5 m) and in the organic system the plot was 13.47 m^2^ (7.7 m x 1.75 m). A 40 cm gap was applied between the plots to avoid contamination of the control objects with biostimulant. Applied biostimulant contained 12.8% of total nitrogen in organic form and about 80% of free amino acids, including about 8% Asp, 12% Glu, 15% Ser, 8% Gly, 1% His, 6% Arg, 5% Thr, 5% Ala, 13% Pro, 0.5% Tyr, 6% Val, 0.5% Met, 3%Ile, 8% Leu, 6% Phe, 2% Lys, 1% Cys. The biostimulant was applied twice during the growing season (the first application at stem elongation stage (BBCH 32) and the second at the beginning of ear emergence (BBCH 53), in rates of 0.5 kg/ha each, in the form of a 0.17% final concentration of the applied solution. The working liquid was sprayed manually, using a plots backpack sprayer with pressure regulator. Field experiments were conducted in 2021, in a conventional and organic systems, in the central part of Poland. Conventional experiments were located in Minikowo (53°10′02″N, 17°44′22″E), in soil with pH 4.7 and the following content of absorbable forms of nutrients: 7.1 mg P_2_O_5_/100 g of soil, 17.1 mg K_2_O/100 g of soil, 4.5 mg MgO/100 g of soil. The experiment in the organic system was located in Luchowo (53°15′40″N, 17°16′26″E), in soil with pH 7.7 and the content of 23.7 mg P_2_O_5_/100 g of soil, 19.7 mg K_2_O/100 g of soil, 4.2 mg MgO/ 100 g of soil. The soil under the experiment was characterized by a very low mineral nitrogen content. In the conventional system, the soil contains 25.0 kg N_min_/ha in a layer 0–30 cm, while in the organic system it is 23.8 kg N_min_/ha in a layer 0–30 cm. Barley sowing in the conventional system was made between the 21st and 31st of March, and in the organic system between the 1st and 10th of April. The sowing density in both experiments was 350 grains/m^2^ and the row spacing was 12.5 cm. No cultivation practices were performed in the organic system during growth (except for the biostimulant application in selected combinations). The conventional system, however, involved the use of typical barley cultivation technology with mineral fertilization and chemical protection. The seed was treated with triticonazole and prochloraz seed dressing in a dose of 4 g and 12 g a.i./100 kg of grain. Pre-sowing fertilization included: N- 70 kg/ha; P_2_O_5_−40 kg/ha; K_2_O- 70 kg/ha. At the stem elongation stage, a mixture of 2,4-D, florasulam and pinoxaden (180 + 3.75 + 40 g a.i./ha) was applied to protect against weeds. Protection against fungal diseases included two treatments–at the first nodule stage (BBCH 31) tiophanate-methyl (700 g a.i./ha) and at the flag leaf sheath thickening (BBCH 41–49) prothioconazole and tebuconazole (125 + 125 g a.i./ha). At the flag leaf stage, cypermethrin (25 g a.i./ha) was used to control the cereal leaf beetle. Harvest was made between the 21st and 31st July, at the full maturity stage, with grain moisture below 14%.

### Determination of chlorophylls

Grain chlorophyll content was determined using the method describe earlier [18]. Chlorophyll extracts were obtained from ground grains (5 g), which were triturated with a mixture of ethanolic solution (70%). Then, after separation of the plant tissue by filtration and centrifugation, the separation of chlorophylls from the remaining dyes was carried out on solid-phase extraction (SPE) columns. The extract prepared in this way was concentrated in a vacuum evaporator at 35°C, dissolved in 1 ml of methanol (Merck) and subjected to chromatographic analysis. Chlorophyll a and chlorophyll b were determined using Acquity UPLC (Waters, USA) with a Waters Acquity PDA detector (Waters, USA). Chromatographic separation was performed on an Acquity UPLC® BEH C18 column (100 mm x 2.1 mm, particle size 1.7 μm) (Waters, Ireland). Elution was carried out using solvent A–methanol and water B. A gradient (mixture of acetonitrile 0.2% (v/v) HCOOH in H_2_O) was used as the eluting phase was used at a flow of 0.4 ml/min. The column and samples were thermostated, the column temperature was 30°C, the test temperature was 10°C. During the analysis, the solutions were degassed in the Waters apparatus. The compounds were identified based on spectra ranging from 200 to 600 nm and retention times compared to standards.

### Determination of anthocyanidins

Grain anthocyanidins content was determined using the method describe earlier [19]. Anthocyanidin extracts were obtained from ground seeds (5 g) which were triturated with a mixture of ethanolic solution (70%) and acetone (1:1). Then, after separation of the plant tissue by filtration and centrifugation, anthocyanidins were separated from the remaining dyes on SPE columns. The extract prepared in this way was concentrated in a vacuum evaporator at 35°C, dissolved in 1 ml of methanol (Merck) and subjected to chromatographic analysis. The following compounds were analysed: delphinidin, luteolinidin, malvidin, peonidine, petunidin, rosinidin, aurantinidin, cyanidin. Anthocyanidins were determined using Acquity UPLC (Waters, USA) with a Waters Acquity PDA detector (Waters, USA). Chromatographic separation was performed on an Acquity UPLC® BEH C18 column (150 mm x 2.5 mm, particle size 1.8 μm) (Waters, Ireland). Elution was carried out using solvent A—methanol, water B and acetonitrile C. A mixture of acetonitrile 0.2% (v/v) HCOOH in H_2_O (gradient) was used as the eluting phase. A gradient was used at a flow of 0.4 ml/min. The column and samples were thermostated, the column temperature was 30°C, the test temperature was 10°C. During the analysis, the solutions were degassed in the Waters apparatus. The compounds were identified on the basis of spectra in the range of 200 to 600 nm. and retention times compared to standards.

### Phytomelanin analysis

The analysis of phytomelanin content was carried out based on the analytical protocol proposed by Merck (https://www.sigmaaldrich.com), with own modification. Phytomelanin extracts were obtained from ground seeds (5 g), which were triturated with a water solution of ethyl alcohol (60%). Then, after separation of the plant tissue by filtration and centrifugation, the separation of phytomelanin from the remaining dyes was carried out on SPE columns. The extract prepared in this way was concentrated in a vacuum evaporator at 35°C, dissolved in 1 ml of methanol (Merck) and subjected to chromatographic analysis. Phytomelanin was determined using Acquity UPLC (Waters, USA) with a Waters Acquity PDA detector (Waters, USA). Chromatographic separation was performed on an Acquity UPLC® BEH C18 column (250 mm x 2.5 mm, particle size 1.5 μm) (Waters, Ireland). Elution was carried out using solvent A—methanol, water B and acetonitrile C. A gradient was used at a flow of 0.4 ml/min. The compound was identified by retention times compared to standards.

### Antiradical capacity

Free radical scavenging using ABTS^+^ (2,2’-azino-bis(3-ethylbenzothiazoline-6-sulfonic acid)) stable radical was performed according to a modification of the improved ABTS^+^ method described by Zhou et al. [20]. The spectrophotometric assay was performed in 96-well plates. Extract (10 μl) or ethanol (10 μl, control) was added to 195 μl of the ABTS^+^ radical solution and left for 30 min until a stable absorbance reading was obtained. The decrease in absorbance at 734 nm was measured relative to the blank test (ethanol).

### Determination of phenolic acids

Contents of 10 phenolic acids: gallic acid (GA), 2.5- dihydroxobenzoic acid (2.5-DHBA), 4-dihydroxobenzoic acid (p-HBA), protocatechuic acid (PCA), syringic acid (SYA), p-coumaric acid (p-CA), chlorogenic acid (CGA), caffeic acid (CA), synaptic acid (SA), ferulic acid (FA), were determined as described by Stuper-Szablewska et al. [21]. Ground grain samples of 0.2 g were subjected to alkaline hydrolysis in 4 mL of 2M aqueous sodium hydroxide solution, followed by acid hydrolysis in 2 mL of 6M aqueous hydrochloric acid solution. Phenolic acids were extracted from the inorganic phase with diethyl ether (2 x 2ml). Acid hydrolysis was then carried out on the ether extracts in 3 mL of 6M aqueous hydrochloric acid solution. The resulting ether extracts were evaporated to dryness in a stream of nitrogen and then dissolved in 1ml of methanol. Chromatographic analysis was performed using a Waters SDS 501 (Waters, Milford, MA, USA) high-performance liquid chromatograph with a Waters 486 Tunable Absorbance Detector (Waters, Milford, MA, USA). Chromatographic separation was performed on an RP C-18, 250 x 4 mm x 5 μm column. A mixture of acetonitrile 0.2% (v/v) HCOOH in H_2_O (gradient) was used as the eluting phase. Measurement was performed at wavelength λ = 320 and 280 nm. Identification of the compounds consisted of comparing the retention time of the tested peak with that of the standard (Sigma‐Aldrich, Poznan, Poland). Analysed only bound fraction of phenolic acids.

### Determination of flavonoids

Composition of flavonoids was determined using a method described by [22]. The extracted flavonoids were separated and identified by an Agilent UPLC equipped with a Nova‐Pak C18 reversed‐phase column (3.9 × 150 mm, 5‐μm particle size; both from Waters, Milford, MA, USA). Solvent A used was 0.3% (v/v) HCOOH in H_2_O, while solvent B used was acetonitrile of HPLC purity grade. The flow rate of the solvents was maintained at 1 mL/min. The gradient profile was as follows: 85% of A at 0 min and 25% of A at 40 min. The mobile phase of the gradient elution was as follows: A, acetonitryl with 0.1% formic acid; and B, 1% aqueous formic acid mixture (pH = 2). Chromatograms were recorded using a UV–Vis detector at λ = 370 nm. The separated compounds were identified based on retention time mapping using a set of standards. The quantity of the following flavonoids was determined using standard solutions (0.001–0.01 μg/mL) of individual compounds (Sigma‐Aldrich, Poznan, Poland). Was determined of seven flavonoids: naringenin, vitaxin, rutin, quercetin, apigenin, kaempferol and lutein.

### Statistical analysis

The results were subjected to multivariate ANOVA statistical analysis. according to a fixed model with three experimental errors appropriate for the split-block system. Differences between means were compared using Tuckey’s HSD test at p = 0.05. Pearson’s simple correlation analysis was also performed. Hierarchical cluster analysis and principal component analysis (PCA) were also performed. All calculations were performed using the statistical package Statistica PL 13.0 (StatSoft, Cracow, Poland).

## Results

### Composition of phenolic acids

Among the phenolic acids identified in the analysed grain, ferulic acid was dominant. Syringic, caffeic and 2.5-hydroxybenzoic acids were present in significantly lower amounts, while sinapic and 4-hydroxybenzoic acids were the least present (Tables 1 and 2).

**Table 1:** Content of phenolic acids—derivatives of hydroxybenzoic acid in barley grain.

Farming System (FS)	Genotype (G)	Treatment (T)	GA mg/kg	2,5-DHBA mg/kg	p-HBA mg/kg	PCA mg/kg	SYA mg/kg
Con	*H*. *v*. *nigricans*	B	10.2^d^ ±0.58	40.1^cd^ ±1.50	15.4^a-c^ ±0.82	18.4^cd^ ±2.16	51.3^b^ ±0.88
		C	12.9^d^ ±0.12	44.5^b-d^ ±2.63	15.3^a-c^ ±0.70	5.9^ef^ ±0.61	49.4^b^ ±1.04
	*H*. *v*. *rimpaui*	B	18.7^c^ ±0.36	65.1^a^ ±3.09	18.2^a^ ±1.94	16.2^c-e^ ±1.83	64.4^ab^ ±11.38
		C	12.3^d^ ±0.93	46.0^b-d^ ±1.72	11.7^bc^ ±1.68	21.3^bc^ ±1.02	60.4^ab^ ±1.05
	*H*. *v*. ’Soldo’	B	26.2^ab^ ±0.95	31.5^de^ ±0.99	15.4^a-c^ ±1.33	5.3^f^ ±0.46	6.6^c^ ±0.40
		C	16.7^c^ ±1.47	20.5^e^ ±1.03	10.9^c^ ±0.34	3.6^f^ ±0.39	5.8^c^ ±0.53
Org	*H*. *v*. *nigricans*	B	18.1^c^ ±0.23	46.4^a-d^ ±0.73	16.1^ab^ ±0.25	31.2^ab^ ±0.36	54.4^b^ ±2.07
		C	18.9^c^ ±0.76	51.6^a-c^ ±2.17	16.6^ab^ ±0.66	34.0^a^ ±1.41	58.9^ab^ ±1.88
	*H*. *v*. *rimpaui*	B	26.4^ab^ ±1.53	61.4^ab^ ±15.01	18.3^a^ ±3.88	38.1^a^ ±11.88	76.1^a^ ±16.98
		C	16.9^c^ ±2.50	53.9^a-c^ ±2.97	14.2^a-c^ ±2.87	34.0^a^ ±0.60	66.4^ab^ ±4.74
	*H*. *v*. ’Soldo’	B	29.6^a^ ±1.53	38.0^de^ ±2.71	15.5^a-c^ ±1.34	7.4^ef^ ±0.95	8.9^c^ ±0.95
		C	23.34^b^ ±1.64	31.1^de^ ±11.60	16.3^ab^ ±2.67	8.0^d-f^ ±1.03	6.8^c^ ±1.37
Con			16.2^b^ ±5.52	41.3^b^ ±14.22	14.5^a^ ±2.76	11.8^b^ ±7.31	39.6^b^ ±25.21
Org			22.2^a^ ±4.96	47.1^a^ ±12.36	16.2^a^ ±2.34	25.4^a^ ±13.71	45.2^a^ ±28.76
	*H*. *v*. *nigricans*		15.0^c^ ±3.81	45.7^b^ ±4.60	15.8^a^ ±0.78	22.4^b^ ±11.74	53.5^b^ ±4.00
	*H*. *v*. *rimpaui*		18.6^b^ ±5.50	56.6^a^ ±10.20	15.6^a^ ±3.73	27.4^a^ ±10.70	66.8^a^ ±10.80
	*H*. *v*. ’Soldo’		24.0^a^ ±5.12	30.3^c^ ±8.30	14.5^a^ ±2.62	6.1^c^ ±1.93	7.0^c^ ±1.42
		B	22.0^a^ ±6.07	47.1^a^ ±13.75	16.5^a^ ±2.12	19.4^a^ ±12.91	43.6^a^ ±28.24
		C	16.4^b^ ±4.59	41.3^b^ ±12.87	14.2^b^ ±2.70	17.8^a^ ±13.13	41.3^a^ ±26.05
p-value	FS		0.0003	0.0009	0.1185	0.0001	0.0581
	G		0.0000	0.0000	0.2145	0.0000	0.0000
	T		0.0000	0.0046	0.0008	0.0061	0.2136
	FS x G x T		0.0106	0.4458	0.2177	0.0001	0.4565

FS–farming system, G–genotype, T–treatment, GA–gallic acid, 2.5–DHBA– 2.5- dihydroxobenzoic acid, p-HBA—4-dihydroxobenzoic acid, PCA–protocatechuic acid, SYA–syringic acid, Org/Con–two farming systems (Org–organic farming, Con–conventional farming), B/C–two biostimulant treatments (B–Biostimulant, C–control); a-f–mean values in column with common letters are not significantly different (ANOVA at the significance level p = 0.05); data = mean ± SD (standard deviation).

**Table 2:** Content of phenolic acids—derivatives of hydroxycinnamic acid in barley grain.

FS	G	T	p-CA mg/kg	CGA mg/kg	CA mg/kg	SA mg/kg	FA mg/kg
Con	*H*. *v*. *nigricans*	B	26.5^a^ ± 0.69	14.8^cd^ ±0.36	61.1^c^ ±0.83	7.00^b-d^ ±0.61	889^c^ ±38.2
		C	21.4^a^ ±0.94	13.1^cd^ ±0.86	15.7^e^ ±0.84	1.96^f^ ±0.55	677^d^ ±24.4
	*H*. *v*. *rimpaui*	B	30.3^a^ ±1.02	17.4^bc^ ±3.71	123^a^ ±3.17	7.72^b-f^ ±1.52	1010^bc^ ±1.5
		C	29.6^a^ ±0.64	15.3^cd^ ±2.43	11.5^e^ ±0.91	13.7^b^ ±1.67	555^de^ ±59.6
	*H*. *v*. ’Soldo’	B	32.1^a^ ±5.71	23.7^ab^ ±3.07	31.1^d^ ±2.97	2.16^f^ ±0.36	392^f^ ±3.7
		C	25.5^a^ ±0.84	10.3^d^ ±0.94	31.0^d^ ±5.67	12.4^b-d^ ±1.08	415^ef^ ±6.6
Org	*H*. *v*. *nigricans*	B	29.4^a^ ±0.32	15.8^cd^ ±0.26	74.6^b^ ±5.86	5.46^ef^ ±0.46	1146^b^ ±80.6
		C	31.1^a^ ±1.32	16.3^cd^ ±0.64	18.7^e^ ±0.75	4.63^ef^ ±0.42	948^c^ ±23.7
	*H*. *v*. *rimpaui*	B	35.3^a^ ±11.5	17.9^bc^ ±3.84	121^a^ ±4.19	21.1^a^ ±3.19	1686^a^ ±27.3
		C	31.6^a^ ±0.68	13.9^cd^ ±2.92	16.6^e^ ±2.54	6.59^d-f^ ±4.54	922^c^ ±74.6
	*H*. *v*. ’Soldo’	B	34.6^a^ ±3.40	30.0^a^ ±0.68	38.3^d^ ±0.84	9.29^b-e^ ±0.94	633^d^ ±37.1
		C	37.1^a^ ±12.3	24.5^a^ ±1.05	35.6^d^ ±1.08	12.8^bc^ ±1.40	599^d^ ±72.8
Con			27.6^b^ ±4.21	15.7^b^ ±4.68	45.6^b^ ±39.2	7.49^b^ ±4.72	657^b^ ±239
Org			33.3^a^ ±6.48	19.7^a^ ±6.08	50.8^a^ ±37.9	9.98^a^ ±6.19	989^a^ ±378
	*H*. *v*. *nigricans*		27.3^b^ ±4.14	14.98^b^ ±1.38	42.5^b^ ±27.0	4.77^c^ ±1.97	915^b^ ±179
	*H*. *v*. *rimpaui*		31.8^a^ ±5.44	16.09^b^ ±3.26	68.1^a^ ±56.5	12.3^a^ ±6.55	1043^a^ ±429
	*H*. *v*. ’Soldo’		32.3^a^ ±7.46	22.11^a^ ±7.71	34.0^c^ ±4.3	9.15^b^ ±4.56	510^c^ ±117
		B	31.4^a^ ±5.54	19.91^a^ ±5.88	74.8^a^ ±37.4	8.80^a^ ±6.26	959^a^ ±422
		C	29.5^a^ ±6.66	15.55^b^ ±4.76	21.5^b^ ±9.22	8.67^a^ ±4.96	686^b^ ±202
p-value	FS		0.0008	0.0147	0.0068	0.0009	0.0000
	G		0.0916	0.0014	0.0197	0.0000	0.0000
	T		0.1285	0.0000	0.0000	0.9219	0.0000
	FS x G x T		0.3222	0.0037	0.0151	0.0001	0.0085

FS–farming system, G–genotype, T–treatment, p-CA–p-coumaric acid, CGA–chlorogenic acid, CA–caffeic acid, SA–synaptic acid, FA–ferulic acid, *H*. v. *nigricans* / *H*. v. *rimpaui* / *H*. v. ’Soldo’–three genotypes of barley, Org–organic farming, Con–conventional farming, B–biostimulant, C–control; a-f–mean values in column with common letters are not significantly different (ANOVA at the significance level p = 0.05).

*H*. *v*. *rimpaui* grain was characterized by the highest content of 2,5-hydroxybenzoic, protocatechuic, syringic, caffeic, sinapic and ferulic acids among the tested genotypes. *H*. *v*. *nigricans*, compared to *H*. *v*. *rimpaui*, contained statistically significantly less of these phenolic acids (by 19.3%, 18.3%, 19.9%, 37.5%, 61.2% and 12.3%, respectively). Genotypes with black coloured grain contained nearly twice as much 2,5-hydroxybenzoic acid, protocatechuic acid, caffeic acid and ferulic acid, and more than eight times as much syringic acid than *H*. *vulgare* ’Soldo’ (Tables 1 and 2). On the other hand, the modern cultivar of barley contained significantly more gallic and chlorogenic acids.

The content of the analysed phenolic acids (with the exception of 4-dihydroxobenzoic acid) was significantly higher in grain from the organic cultivation system compared to the conventional one (Tables 1 and 2). The highest increase in the content of phenolic acids in organic grain was recorded for syringic, ferulic and protocatechuic acids, by 32.2%, 50.6% and 116.1%, respectively. However, the response to the farming system varied according to the genotype. The cultivar ’Soldo’ accumulated significantly more ferulic acid in grain under organic cultivation conditions, while *H*. *v*. *nigricans* and *H*. *v*. *rimpaui* accumulated more protocatechuic acid in comparison to conventional cultivation (Tables 1 and 2).

The content of phenolic acids in the grain was also influenced by the foliar application of a biostimulant based on amino acids during the barley growing period (Tables 1 and 2). On average, for the studied genotypes, grain after biostimulant application contained significantly more gallic, 2,5-hydroxybenzoic, 4-hydroxybenzoic, chlorogenic, caffeic and ferulic acids (by 32.4%, 14.2%, 16.3%, 28.0%, 24.7% and 39.8%, respectively) compared to the control sample. The response to the biostimulant was different in barley genotypes. Both in the organic and conventional cultivation systems, the use of the biostimulant resulted in an increase in the concentration of gallic acid in the cultivar ’Soldo’, and of caffeic and ferulic acid in the primary genotypes of barley (*H*. *v*. *nigricans* and *H*. *v*. *rimpaui)*. The biostimulant had a beneficial effect on the concentration of sinapic acid in the grain of *H*. *v*. *nigricans* only in conventional cultivation, while *H*. *v*. *rimpaui* in organic cultivation.

### Composition of flavonoids

The dominant flavonoids in grains of *H*. *v*. *nigricans* and *H*. *v*. *rimpaui* were naringenin, quercetin, apigenin, kaempferol and luteolin. Among the flavonoids determined in grains of *H*. *v*. ’Soldo’, the most abundant was vitexin (Table 3). The tested genotypes were similar only in terms of rutin content. The grain of *H*. *v*. *rimpaui* compared to *H*. *v*. *nigricans* was characterized by a significantly higher concentration of naringenin, vitexin and apigenin (by 37.6%, 38.5%, 52.1%, respectively) and significantly lower concentration of quercetin and kaempferol (by 13.2% and 9%, respectively).

**Table 3:** Content of flavonoids in barley grain.

FS	G	T	NGN mg/kg	VIT mg/kg	RU mg/kg	QU mg/kg	API mg/kg	KPF mg/kg	LU mg/kg
Con	*H*. *v*. *nigricans*	B	175.4^de^ ±6.16	2.8^f^ ±0.92	19.2^a^ ±0.68	145.9^b-d^ ±7.6	14.7^d-ef^ ±2.1	37.0^cd^ ±1.3	31.9^cd^ ±1.4
		C	159.7^e^ ±8.6	4.6^ef^ ±0.44	16.8^a^ ±0.47	122.3^d^ ±2.6	30.4^c^ ±1.0	17.6^ef^ ±1.3	29.5^cd^ ±1.0
	*H*. *v*. *rimpaui*	B	270.5^ab^ ±23.7	16.4^bc^ ±3.95	16.5^a^ ±6.06	124.6^d^ ±3.5	77.6^a^ ±1.6	15.5^ef^ ±0.6	33.7^c^ ±2.9
		C	193.8^c-e^ ±8.8	6.7^ef^ ±0.30	16.2^a^ ±1.01	126.4^d^ ±9.5	28.8^cd^ ±1.4	7.2^f^ ±1.2	17.4^de^ ±0.8
	*H*. *v*. ’Soldo’	B	28.4^f^ ±1.2	13.3^cd^ ±0.80	22.0^a^ ±1.56	28.2^e^ ±3.4	3.5^f^ ±0.3	18.0^ef^ ±1.7	1.8^ef^ ±0.1
		C	14.9^f^ ±2.2	10.0^de^ ±0.80	15.6^a^ ±2.20	15.7^e^ ±3.9	5.2^ef^ ±1.1	24.1^de^ ±3.0	1.1^f^ ±0.03
Org	*H*. *v*. *nigricans*	B	209.6^cd^ ±9.0	17.0^bc^ ±0.24	17.9^a^ ±0.23	181.1^a-c^ ±4.3	55.5^b^ ±1.0	47.5^bc^ ±0.8	55.8^b^ ±1.0
		C	183.6^de^ ±4.4	17.6^bc^ ±0.70	18.7^a^ ±0.75	206.7^a^ ±8.9	62.0^ab^ ±2.6	52.8^ab^ ±2.2	62.4^ab^ ±2.6
	*H*. *v*. *rimpaui*	B	304.5^a^ ±18.8	19.7^b^ ±4.03	17.8^a^ ±9.17	133.2^cd^ ±24.6	75.3^a^ ±16.9	63.1^a^ ±15.2	75.8^a^ ±16.9
		C	232.9^bc^ ±28.7	15.4^b-d^ ±2.71	16.6^a^ ±2.53	185.2^ab^ ±49.2	65.7^ab^ ±4.6	55.3^ab^ ±3.2	66.1^ab^ ±4.7
	*H*. *v*. ’Soldo’	B	45.3^f^ ±3.5	67.0^a^ ±1.61	23.3^a^ ±1.80	32.9^e^ ±1.4	17.5^c-f^ ±1.6	36.4^cd^ ±1.6	5.8^ef^ ±1.0
		C	41.5^f^ ±12.5	55.8^b^ ±3.31	20.9^a^ ±8.05	22.6^e^ ±9.2	19.4^c-e^ ±1.2	19.4^ef^ ±3.4	4.1^ef^ ±0.8
Con			140.4^b^ ±94.1	9.0^b^ ±5.14	17.7a ±3.26	93.8^b^ ±53.3	26.7^b^ ±25.8	19.9^b^ ±9.5	19.2^b^ ±14.1
Org			169.6^a^ ±100.1	32.1^a^ ±21.73	19.2a ±4.90	127.0^a^ ±78.1	49.2^a^ ±24.0	45.8^a^ ±15.7	45.0^a^ ±30.4
	*H*. *v*. *nigricans*		182.1^b^ ±19.8	10.5^c^ ±7.14	18.2^a^ ±1.05	164.0^a^ ±34.2	40.7^b^ ±20.0	38.8^a^ ±14.1	44.9^a^ ±15.1
	*H*. *v*. *rimpaui*		250.4^a^ ±46.9	14.6^b^ ±5.69	16.8^a^±4.87	142.3^b^ ±35.3	61.9^a^ ±21.8	35.3^b^ ±26.2	48.3^a^ ±25.9
	*H*. *v*. ’Soldo’		32.5^c^ ±13.7	36.5^a^ ±26.38	20.5^a^ ±4.80	24.9^c^ ±8.4	11.4^c^ ±7.5	24.5^c^ ±7.9	3.2^b^ ±2.0
		B	172.3^a^ ±108.0	22.7^a^ ±21.22	19.4^a^ ±4.60	107.6^a^ ±59.7	40.7^a^ ±31.4	36.3^a^ ±17.68	34.15^a^ ±27.4
		C	137.7^b^ ±83.9	18.4^b^ ±17.90	17.5^a^ ±3.55	113.2^a^ ±77.1	35.3^b^ ±22.6	29.4^b^ ±18.80	30.10^b^ ±26.7
p-value	FS		0.0021	0.0000	0.2128	0.0041	0.0004	0.0001	0.0000
	G		0.0000	0.0000	0.2242	0.0000	0.0001	0.0000	0.0000
	T		0.0000	0.0056	0.1791	0.7777	0.0466	0.0000	0.0001
	FS x G x T		0.4897	0.0029	0.7446	0.3001	0.0018	0.0000	0.0422

FS–farming system, G–genotype, T–treatment, NGN–naringenin, VIT–vitaxin, RU–rutin, QU—quercetin, API–apigenin, KPF–kaempferol, LU–luteolin, *H*. v. *nigricans* / *H*. v. *rimpaui* / *H*. v. ’Soldo’–three genotypes of barley, Org–organic farming, Con–conventional farming, B–biostimulant, C–control; a-f–mean values in column with common letters are not significantly different (ANOVA at the significance level p = 0.05).

On average for genotypes, grains from the organic system contained significantly more naringenin by 20.7%, quercetin by 35.3%, apigenin by 84.4%, kaempferol by 129%, luteolin by 134% and vitexin by 256.8% compared to the conventional system. However, different genotypes responded differently to farming systems. In the modern cultivar ’Soldo’, the organic cultivation system favoured the concentration of vitexin, in *H*. *v*. *nigricans*, apigenin and luteolin, and in the genotype of *H*. *v*. *rimpaui*, kaempferol (Table 3).

The application of the biostimulant, on average for the tested genotypes, significantly increased the content of most flavonoids (luteolin by 13.5%, apigenin by about 15.3%, vitexin by 20.2%, kaempferol by 23.2% and naringenin by 25.1%) (Table 3). The response, however, differed by genotype and farming system. *H*. *v*. *nigricans* in organic cultivation did not show any response with the flavonoid content to the biostimulant application, while in the conventional system the application of the preparation significantly increased the content of kaempferol. In contrast, the *H*. *vulgare* cultivar ’Soldo’ showed a positive response with flavonoid concentration (vitexin and kaempferol) to a biostimulant only in organic cultivation. *H*. *v*. *rimpaui* showed the strongest response to the biostimulant. In the conventional system, it caused an increase in the concentration of naringenin (by 39.6%), vitexin (2.5 times), apigenin (2.7 times) and luteolin (1.9 times), and in the organic system, only naringenin (by 30.7%).

### Plant pigments

Chlorophyll a and chlorophyll b (Table 4) were present only in the grains of black-grained cultivars, and their concentration was significantly higher in the *H*. *v*. *nigricans* genotype. Organic farming had a positive effect on the average concentration of chlorophyll b and a negative effect on the content of chlorophyll a. The biostimulant application significantly increased the content of chlorophyll b while reducing the content of chlorophyll a in grains.

**Table 4:** Content of plant colour substances in grain and antioxidant activity (ABTS^+^) of barley.

FS	G	T	Chl a	Chl b	Dph	Lut	PhM	ABTS^+^
			mg/kg	mg/kg	mg/kg	mg/kg	mg/kg	%
Con	*H*. *v*. *nigricans*	B	0.65^d^ ±0.04	0.08^b^ ±0.01	1.17^de^ ±0.02	0.26^cd^ ±0.02	149.6^b^ ±5.82	90.1^a^ ±0.49
		C	2.87^a^ ±0.07	0.00^c^	1.26^c-e^ ±0.02	0.52^b^ ±0.02	34.7^d^ ±4.19	87.3^a^ ±2.54
	*H*. *v*. *rimpaui*	B	0.82^d^ ±0.05	0.06^b^ ±0.01	1.72^b^ ±0.05	0.17^d^ ±0.02	129.0^c^ ±4.12	79.4^a^ ±11.52
		C	2.14^c^ ±0.03	0.00^c^ ±0.00	1.07^e^ ±0.03	0.16^d^ ±0.02	46.0^d^ ±3.18	86.9^a^ ±1.92
	*H*. *v*.’Soldo’	B	0.00^d^	0.00^c^	0.00^f^	0.00^e^	0.00^e^	61.4^a^ ±2.77
		C	0.00^d^	0.00^c^	0.00^f^	0.00^e^	0.00^e^	72.7^a^ ±5.21
Org	*H*. *v*. *nigricans*	B	0.56^d^ ±0.09	0.12^a^ ±0.02	1.37^cd^ ±0.10	0.22^cd^ ±0.02	171.9^a^ ±5.76	87.8^a^ ±1.75
		C	2.66^ab^ ±0.48	0.00^c^ ±0.00	1.39^cd^ ±0.20	0.64^a^ ±0.04	40.7^d^ ±7.01	86.7^a^ ±6.68
	*H*. *v*. *rimpaui*	B	0.47^de^ ±0.03	0.11^a^ ±0.02	2.27^a^ ±0.18	0.28^c^ ±0.08	163.5^a^ ±3.32	89.8^a^ ±0.73
		C	2.19^bc^ ±0.14	0.00^c^ ±0.00	1.48^bc^ ±0.16	0.26^cd^ ±0.08	39.7^d^ ±2.46	90.6^a^ ±0.37
	*H*. *v*.’Soldo’	B	0.00^d^	0.00^c^	0.00^f^	0.00^e^	1.2^e^ ±0.10	58.3^a^ ±13.00
		C	0.00^d^	0.00^c^	0.00^f^	0.00^e^	0.00^e^	75.8^a^ ±4.16
Con			1.08^a^ ±1.10	0.02^b^ ±0.03	0.87^b^ ±0.67	0.18^b^ ±0.18	59.9^b^ ±60.7	79.6^a^ ±11.30
Org			0.98^b^ ±1.10	0.04^a^ ±0.06	1.08^a^ ±0.87	0.23^a^ ±0.22	69.5^a^ ±73.5	81.5^a^ ±12.92
	*H*. *v*. *nigricans*		1.68^a^ ±1.15	0.05^a^ ±0.06	1.30^b^ ±0.13	0.41^a^ ±0.18	99.2^a^ ±65.0	88.0^a^ ±3.43
	*H*. *v*. *rimpaui*		1.40^b^ ±0.81	0.04^b^ ±0.05	1.63^a^ ±0.46	0.22^b^ ±0.07	94.5^a^ ±55.6	86.7^a^ ±6.80
	*H*. *v*.’Soldo’		0.00^c^	0.00^c^	0.00^c^	0.00^c^	0.3^b^ ±0.54	67.0^b^ ±9.98
		B	0.42^b^ ±0.32	0.06^a^ ±0.05	1.09^a^ ±0.87	0.15^b^ ±0.12	102.5^a^ ±75.5	77.8^b^ ±14.90
		C	1.64^a^ ±1.23	0.00^b^ ±0.00	0.87^b^ ±0.65	0.26^a^ ±0.25	26.8^b^ ±20.1	83.3^a^ ±7.62
p-value	FS		0.0031	0.0002	0.0060	0.0214	0.0040	0.5234
	G		0.0000	0.0000	0.0000	0.0000	0.0000	0.0000
	T		0.0000	0.0000	0.0000	0.0000	0.0000	0.0172
	FS x G x T		0.0121	0.0013	0.1968	0.1704	0.0193	0.4378

FS–farming system, G–genotype, T–treatment, Chl a–chlorophyll a, Chl b–chlorophyll b, Dph–delphinidine, Lut–luteolinidine, PhM–phytomelanin, *H*. v. *nigricans* / *H*. v. *rimpaui* / *H*. v. ’Soldo’–three genotypes of barley, Org–organic farming, Con–conventional farming, B–biostimulant, C–control; a-f–mean values in column with common letters are not significantly different (ANOVA at the significance level p = 0.05).

As in the case of chlorophylls, anthocyanin pigments (delphinidin, luteolinidin) were detected only in grains of primary genotypes (Table 4). Both the use of the biostimulant and cultivation in the organic system significantly increased the content of delphinidine in grain. The biostimulant, however, limited the content of luteolinidine in grain.

As in the case of chlorophylls, phytomelanin (Table 4) was found in significant amounts in black grains (*H*. *v*. *nigricans* and *H*. *v*. *rimpaui*). The modern cultivar ’Soldo’, with yellow grain, generally did not contain phytomelanin. Its trace amounts were detected only in grains of this cultivar from conventional cultivation. On average, for the tested genotypes, grain from the organic system contained significantly more phytomelanin (by 16%) than that from the conventional system. The application of an amino acid biostimulant also had a very strong effect on the concentration of this compound in grains. There was 3.8 times more phytomelanin in grain from plants treated with the biostimulant than in the control.

ABTS^+^ antioxidant activity was significantly differentiated depending on barley genotype. The highest ABTS^+^ value was found in black-grained forms, which had about 20 percentage points higher potential for free radical removal than the modern yellow-grained cultivar (Table 4).

### Multivariate analysis

**Fig 2:**
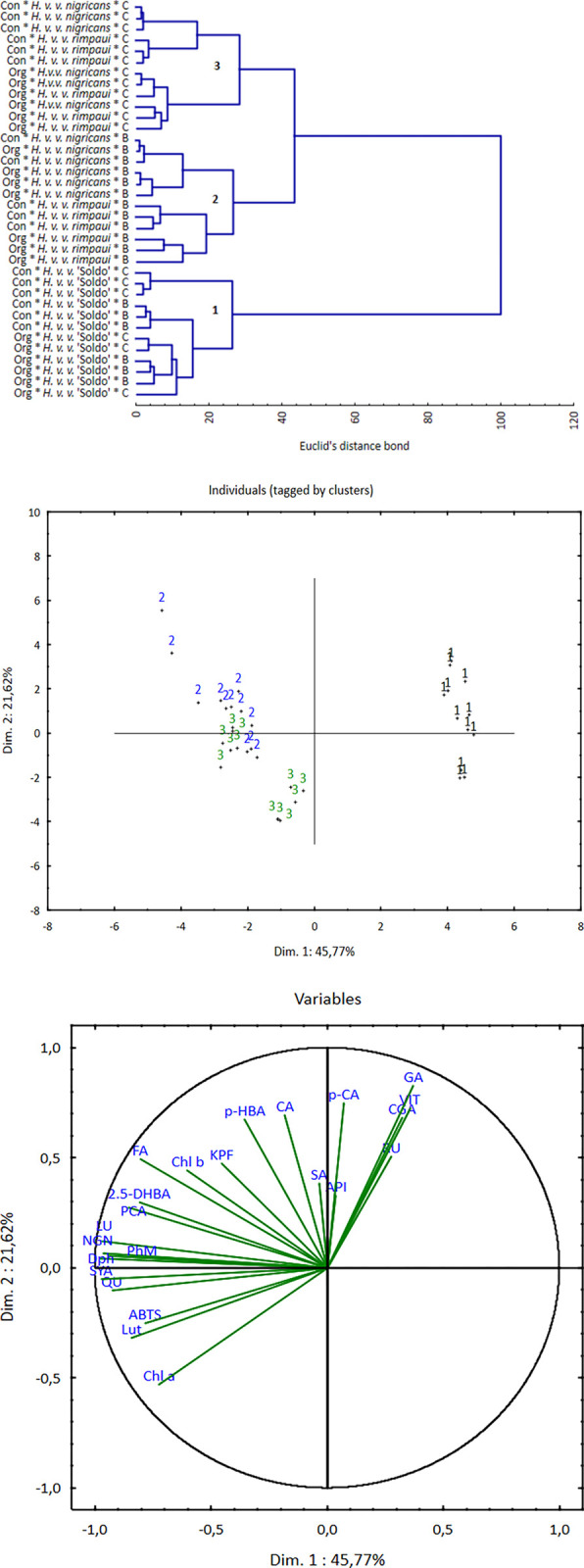
(a) Euclidean distance projection of the tested *H*. *vulgare* L. genotype, (b) principal scatter diagram of the tested parameters, and (c) principal scatter diagram of trials tagged by clusters.

The hierarchical cluster analysis performed divided the analysed cases into 3 clusters (Fig 2A). All combinations with *H*. *vulgare* ’Soldo’ were classified in cluster 1. Cluster 2 was composed of combinations that included *H*. *v*. *nigricans* and *H*. *v*. *rimpaui* treated with the biostimulant. In cluster 3, there were combinations with black-grained genotypes not subjected to biostimulation.

In the analysis of principal components, two components were indicated, describing a total of 67.4% of the variance (Fig 2C). The graphs show the relationships between these components and cases grouped by clusters and qualitative variables. Lines corresponding to positively correlated variables point in the same direction, while lines corresponding to negatively correlated variables point in opposite directions. The length of the lines indicates the effect on the respective components (Fig 2B). The strongest effect on the components was exerted by naringenin, quercetin, lutein, delphinidin, syringic acid, phytomelanin, ferulic acid and gallic acid. Qualitative variables clearly formed two negatively correlated groups with strong, internal dependencies. The first group consisted of gallic acid (GA), chlorogenic acid (CGA), vitexin (VIT) and rutin (RU), while the second group consisted of naringenin (NGN), lutein (LU), quercetin (QU), syringic acid (SYA), phytomelanin (PhM) and delphinidin (Dph). The scatterplot of the cases on the plane of two principal components indicates that the content of GA, CGA, and VIT is related to cluster 1 describing *H*. *vulgare* ’Soldo’. A higher content of NGN, LU, QU, SYA, PhM and DpH was found in cases from clusters 2 and 3 describing black-grained genotypes subjected and not subjected to the application of the amino acid biostimulant. The biostimulant application (cluster 2) resulted in an increased share of phenolic acids (FA, PCA, p-HBA, 2,5-DHBA, CA), kaempferol and chlorophyll b in the grain of primary genotypes.

**Fig 3:**
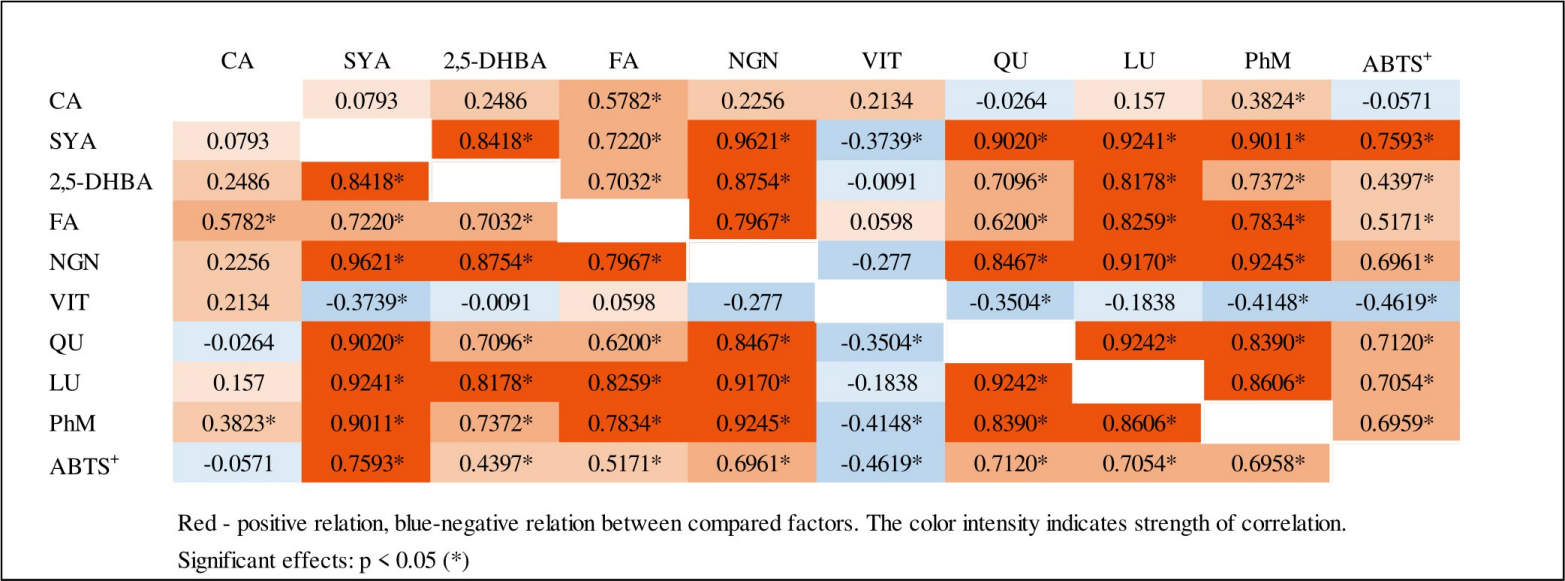
Correlation matrix of phenolic compounds and ABTS^+^. Red—positive relation, blue-negative relation between compared factors. The color intensity indicates strength of correlation. Significant effects: p < 0.05 (*).

Pearson’s simple correlation analysis (Fig 3) showed a very strong correlation with a high coefficient of determination between syringic acid and the most important flavonoids–naringenin (r = 0.9621; r^2^ = 0.9257), quercetin (r = 0.9020; r^2^ = 0.8136) and luteolin (r = 0.9241; r^2^ = 0.8539). Also, the content of phytomelanin strongly depended on syringic acid and naringenin. ABTS^+^ showed the strongest correlation with the content of syringic acid, naringenin, quercetin, luteolin and phytomelanin.

## Discussion

The grain of the tested barley genotypes contained the most phenolic acids derived from hydroxycinnamic acid, in particular ferulic acid. Similarly, other researchers [23, 24] indicate the highest share of ferulic acid in the composition of phenolic acids in barley grains. The synthesis and accumulation of phytochemical compositions in plant tissues are influenced by genotype, growing environment and their interaction [25]. In studies by Jin et al. [26] barley genotypes with black grains were characterized by a significantly higher concentration of total phenolic compounds and the total phenolic acids compared to genotypes with yellow grain. Similarly, in the present study, the profile of phenolic acids depended on the genotype of barley. Gallic and chlorogenic acids were the most abundant in the yellow grains of *H*. *vulgare* cultivar ’Soldo’. Compared to this cultivar, the black-coloured grain of the primary genotypes (*H*. *v*. *nigricans* and *H*. *v*. *rimpaui*) contained from a few to dozen times more phenolic acids, such as 2,5-hydroxybenzoic acid, protocatechuic acid, caffeic acid, ferulic acid and syringic acid. The concentration of flavonoids was also related to the genotype. The grain of the primary genotypes contained more flavonoids, mainly naringenin and quercetin, than the modern yellow barley cultivar. According to Glagoleva et al. [27] the metabolic pathways responsible for the black colour of the grains result from the presence of the Blp gene, which affects the increased synthesis of phenolic compounds (ferulic acid in particular), which, in turn, create black pigmentation of the grains through the oxidation process.

In the present research, apart from genetic factors, the concentration of phenolic acids and flavonoids was also affected by agrotechnical factors. The obtained results clearly indicate the beneficial effect of the organic farming system on the content of phenolic acids in grain. In grains of primary genotypes, the concentration of protocatechuic acid increased, while the *H*. *vulgare* ’Soldo’ responded with an increase in the content of ferulic acid. When analysing the impact of the tested farming systems, attention should also be paid to the differences in the content of available phosphorus and soil reaction in both locations of field experiments. Under conventional farming system, low P content and acidity were observed, while in the organic system the soil was characterized by high P content and alkaline soil reaction. According to many researchers [28–31], the content of phenolic compounds in plants is negatively correlated with the pH of the soil. The acidic reaction of the soil creates a stressful environment for plants, which in turn stimulates the synthesis of phenolic compounds in plants. According to Wright et. al. [32] and Abbas et. al. [33], the P content in the soil does not affect the concentration of phenolic compounds in plants. In turn, Wang et. al. [34] and Corte-Baptistella et. al. [35] reported reduced accumulation of phenolic compounds under high phosphorus availability. These studies would therefore indicate that the soil conditions under conventional farming are more favourable to synthesising phenolic compounds than under organic. Nevertheless, significantly higher content of these metabolites were observed under organic farming system, which may further indicate a beneficial effect of such cultivation methods on the phenolic content. Maver et al. [36], by examining the concentration of secondary metabolites in barley roots proved that phosphorus deficiency results in an increased accumulation of phenylpropanoids, including flavonoids. Differing strategies in soil nutrient management cause soil conditions in organic and conventional farming systems to differ from each other, as indicated by Mader et al. [37], Stockdale et al. [38] and Montgomery and Bikle [39]. These authors unanimously report that the soil in the organic farming system contains significantly more organic matter and microorganisms compared to conventional system, which affects the sorption capacity as well as the transformation and availability of soil nutrients. According to Mader [37] soil with an organic farming system is characterized by a higher activity of dehydrogenase, protease and phosphatase, which accelerates the decomposition of protein and organic phosphorus in the soil, thus affecting the availability of these components for plants. The varied availability of soil nutrients, which differs between the farming systems compared, may therefore be one of the components affecting the content of secondary metabolites in barley grains. In a study by Ostrowska-Kołodziejczak et al. [40], the grain of a cereal mixture of oats, wheat and barley grown in an organic system contained significantly more phenolic acids, in particular ferulic and p-coumaric acids. Effect of organic farming on the content of phenolic compounds in agricultural products became the subject of a meta-analysis conducted by Barański et. al. [41]. These authors, based on 343 peer-reviewed publications, indicated that the concentration of phenolic acids in food from organic farming is 19% higher compared to the conventional system. Higher concentrations of phenolic compounds in organic crops may be the result of increased biotic and abiotic stress occurring in such a farming system, as many phenolic compounds are accumulated by plants in response to stress conditions. Phenolic compounds, including flavonoids, play important biochemical and physiological roles in plant cells, particularly helping to alleviate environmental stress [25]. Expression of the HvPAL gene, which encodes the phenylalanine ammonia enzyme, was increased in salt-tolerant barley, while it was decreased in salt-stress-sensitive barley [42]. According to Przybylska-Balcerek et al. [43], ferulic acid has antioxidant, antibacterial and antifungal properties, and this effect is increased as a result of the synergy of combining this acid with sinapic and gallic acids and some flavonoids. However, there is no unequivocal evidence of the relationship between the pressure of pests and the concentration of polyphenols in crops from organic farming. On the other hand, there is an increasing number of reports proving that the differences in fertilization systems, especially nitrogen fertilization, between the organic and conventional farming systems are an important factor influencing the concentration of phenolic compounds in organic crops [41]. Also, in the present study, there were significant differences in the method of fertilization between the compared cultivation systems. In conventional production, a rate of 60 kg N/ha was applied in the form of ammonium nitrate, while in organic cultivation, no fertilizers, even organic ones, were used before barley cultivation. This resulted in visible signs of nitrogen deficiency during the growing season. Potentially, this could have contributed to the increase in differences in the concentration of phenolic compounds between the farming systems compared. Under conditions of prolonged nitrogen deficiency, there is a significant increase in the activity of L-phenylalanine ammonia lyase (PAL), the enzyme responsible for the catalysis of the reaction of ammonia elimination from aromatic amino acids (phenylalanine and tyrosine) in the phenylpropanoid pathway, leading to the formation of cinnamic acid which is a substrate in the biosynthesis of many phenolic compounds [44].

In the present study, the application of a biostimulant based on amino acids during the barley growing period had a positive effect on the content of phenolic acids and flavonoids in the harvested grain, with the primary genotypes with black grain, especially *H*. *v*. *rimpaui* responding most often. In studies of other species, foliar application of the amino acid phenylalanine increased the activity of L-phenylalanine ammonia lyase PAL and the content of ferulic acid in pea plants [45]. The pathway of phenylpropanoid metabolism and the variety of factors inducing their synthesis in plants is regulated by the expression of multigene families encoding enzymes participating in this pathway—phenylalanine ammonia lyase (PAL) and chalcone synthase (CHS). Various biotic and abiotic factors acting on plants affect the level of transcription of genes encoding enzymes of the phenylpropanoid synthesis pathway [46]. According to Ma et al. [47], varied genotypes of barley under the influence of stress show changes in the level of accumulation of proteins involved in secondary metabolism—mainly phenylalanine ammonia lyase (PAL), isoflavone reductase and caffeic acid *O*-methyltransferase, the enzyme responsible for converting caffeic acid into ferulic acid. Amino acids, in turn, are characterized by high mobility and easy transport in plants. Thanks to this and their biological activity they affect the synthesis of protein substances in plants, which also translates into increased enzyme activity and the production of phenolic compounds [48].

Chlorophyll pigments and anthocyanins were determined only in black-coloured grains (*H*. *v*. *nigricans* and *H*. *v*. *rimpaui*). Dark grains also contained significant amounts of phytomelanin. In addition to genetic factors, agricultural factors also had an impact on the content of this compound. Grain from organic farming contained 16% more of this compound compared to the conventional system. However, the foliar application of an amino acid biostimulant had a much stronger effect on the content of phytomelanin, which increased the content of this compound in *H*. *v*. *nigricans* and *H*. *v*. *rimpaui* grains several times. Phytomelanin is a biopolymer with dark pigmentation, which is widespread among living organisms and gives them a dark colour [8]. Phytomelanins are synthesized by enzymatic oxidation of simple phenolic precursors such as tyrosine, cinnamic acid and cinnamic acid derivatives [49]. Phytomelanin biosynthesis is controlled by the Blp1 gene located in the 0.8 Mb locus on chromosome 1H [27]. The examined agricultural factors affected the content of phytomelanin in a similar way as the concentration of phenolic acids, which seems to confirm the reports on the synthesis of this compound from cinnamic acid derivatives. This claim is also supported by a high correlation coefficient between the content of phytomelanin and the concentration of phenolic acids and flavonoids.

The ABTS^+^ antioxidant activity significantly depended on the genetic factor. The black grain genotypes were characterized by a higher ability to scavenge free radicals than the *H*. *v*. ’Soldo’, which resulted from a much higher concentration of bioactive compounds. A strong positive correlation between the ABTS^+^ antioxidant activity and the concentration of syringic acid, naringenin, quercetin, luteolin and phytomelanin was demonstrated. According to other researchers [49], hydroxycinnamic acids are characterized by higher antioxidant activity than hydroxybenzoic acids. According to Chen et al. [50], in addition to the basic structure, the power of scavenging free radicals by individual phenolic acids is also affected by their structure, specifically the presence of hydroxyl and methoxy groups. In a study by Chen et al. [50], dihydroxy acids (caffeic and protocatechuic acids) had higher antioxidant activity than other phenolic acids, except for the acids with the largest number of methoxyl groups (synapic and syringic acids). According to these authors, in the group of benzoic acid derivatives, the free radical scavenging capacity of syringic acid is tens of thousands of times greater than that of 4-hydroxybenzoic acid. These reports explain the strong correlation between syringic acid and antioxidant activity observed in the present study. Among the phenolic acids found in grains, syringic and caffeic acids are compounds with the highest ability to scavenge free radicals. Only sinapic acid has greater potential, but it was present in grains in an amount several times lower than syringic and caffeic acids. Its particularly high concentration was determined in grains of black-grained genotypes. The grain of these genotypes also contained much more other acids with a strong antioxidant effect (caffeic and protocatechuic acids) than *H*. *v*. ’Soldo’. In the present study, the correlation analysis also showed a strong relationship between the ABTS^+^ antioxidant activity and the content of flavonoids (naringenin, quercetin and luteolin) in grains. Flavonoids are characterized by high antioxidant potential, which results from their chemical structure [51]. Quercetin and luteolin can be indicated as the strongest antioxidants among the compounds detected in the tested barley grains. In a study by Chen et. al. [52] on coloured rice cultivars, antioxidant activity was strongly correlated with naringenin content.

As for phenolic acids and flavonoids, the present study also showed a strong positive relationship between ABTS^+^ and the content of phytomelanin. However, the literature lacks information on the antioxidant properties of this compound. Correlation analysis and principal component analysis (PCA) also showed a relationship between the content of phytomelanin and the contents of syringic acid, quercetin, naringenin and luteolin. It is also known that phytomelanin is formed from oxidized phenolic compounds [49]. It can therefore be assumed that the correlation between the phytomelanin content and the antioxidant activity was related to the concentration and oxidation of the mentioned phenolic compounds. In studies by Yang et al. [26], dark-coloured barley genotypes were characterized by a higher antioxidant potential, which was associated with the content of phenolic compounds. Many authors obtained similar high correlation for antioxidant activity and phenolic compounds content in coloured barley genotypes [53–55]. These results therefore indicate that, apart from the total content of phenolic acids, the composition of phenolic compounds is also of great importance for the antioxidant activity of grain. The similarity of the two tested black-grained genotypes (*H*. *v*. *nigricans* and *H*. *v*. *rimpaui*) and the difference of the modern yellow-grained cultivar in terms of the composition of bioactive compounds and antioxidant activity was also confirmed by the agglomeration cluster analysis. The biostimulant application was an equally strong factor differentiating the values of these variables.

## Conclusions

The analysis of the original genotypes of black grain barley (*H*. *v*. *rimpaui*, and *H*. *v*. *nigricans*) and the modern yellow grain cultivar *(H*. *v*. ’Soldo’) showed the effect of the studied factors: genotype, cultivation system (organic and conventional) and foliar application of the biostimulant on the profile of phenolic compounds and the antioxidant potential of grain. The highest content of most phenolic acids and flavonoids was found in the grain of *H*. *v*. *rimpaui*, followed by *H*. *v*. *nigricans*, and the lowest in *H*. *v*. ’Soldo’. The primary genotypes with black grain were the only ones that contained small amounts of anthocyanin and chlorophyll pigments, while they were distinguished by an extremely high concentration of phytomelanin. Cultivation of barley in an organic system as well as the application of the biostimulant generally had a positive effect on the content of phenolic compounds; however, the interaction of cultivation factors and barley genotype was significant in shaping the content of certain phenolic acids (gallic, protocatechuic, chlorogenic, caffeic, synaptic, and ferulic) and flavonoids (vitexin, apigenin, kaempferol, and luteolin). The strongest positive response with the concentration of phenolic compounds to the application of the biostimulant was shown in cultivars with black grain, mainly *H*. *v*. *rimpaui* in conventional cultivation. The biostimulant stimulated the synthesis of phytomelanins to the greatest extent in black-grain genotypes, cultivated both in a conventional and organic system. ABTS^+^ antioxidant activity of grain of primary barley genotypes was higher than that of the modern cultivar. The ABTS^+^ value was strongly correlated with the content of syringic acid, naringenin, quercetin, luteolin and phytomelanin, which confirms the strong antioxidant properties of these compounds.

## Supporting information

S1 Appendix Comparison of chromatograms of phenolic acids for *H*. *v*. ’Soldo’ (black line), *H v*. v. *nigricans* (blue line) and *H*. *v*. v. *rimpaui* (green line): 1- gallic acid, 2–2,5-dihydroxobenzoic acid, 3–4-dihydroxobenzoic acid, 4—caffeic acid, 5- syringic acid, 6- p-coumaric acid, 7—ferulic acid, 8—protocatechic acid, 9—sinapic acid, 10—chlorogenic acid.
(TIF)

S1 Table Combined analysis of variance based on a fixed effect model for phenolic compounds and antiradical capacity in barley grain from organically and conventionally trials. (DOCX)
