# Supplementary figures and images for: Profile of phenolic compounds and antioxidant activity of organically and conventionally grown black-grain barley genotypes treated with biostimulant
Source: PLoS One. 2023 Jul 12;18(7):e0288428. doi: 10.1371/journal.pone.0288428 (PMC10337966; doi:10.1371/journal.pone.0288428)

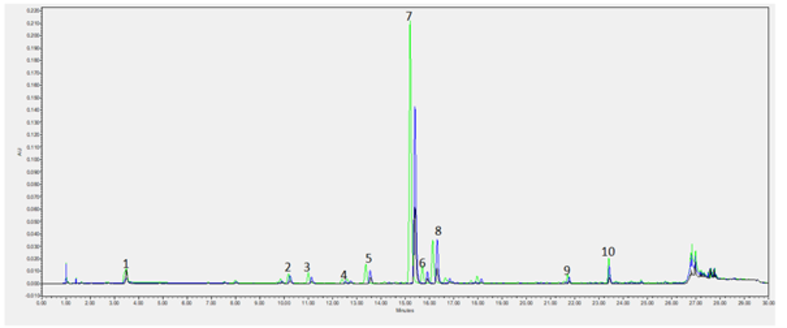

Supplement: S1 Appendix — Comparison of chromatograms of phenolic acids for H. v. ’Soldo’ (black line), H v. v. nigricans (blue line) and H. v. v. rimpaui (green line): 1- gallic acid, 2–2,5-dihydroxobenzoic acid, 3–4-dihydroxobenzoic acid, 4—caffeic acid, 5- syringic acid, 6- p-coumaric acid, 7—ferulic acid, 8—protocatechic acid, 9—sinapic acid, 10—chlorogenic acid. (TIF) [file pone.0288428.s001.tif]
