# Supplementary material for: Profile of phenolic compounds and antioxidant activity of organically and conventionally grown black-grain barley genotypes treated with biostimulant
Source: PLoS One. 2023 Jul 12;18(7):e0288428. doi: 10.1371/journal.pone.0288428 (PMC10337966; doi:10.1371/journal.pone.0288428)
Supplement: S1 Table — (DOCX) [file pone.0288428.s002.docx]

| Parameters | Sources of variation | SS | Df | MS | F | p |
| --- | --- | --- | --- | --- | --- | --- |
| Gallic acid | FS | 8.1261 | 1 | 8.1261 | 140.3200 | 0.0003 |
|  | Error I | 0.2316 | 4 | 0.0579 |  |  |
|  | G | 10.8265 | 2 | 5.4133 | 175.0757 | 0.0000 |
|  | FS x G | 0.4593 | 2 | 0.2296 | 7.4271 | 0.0150 |
|  | Error II | 0.2474 | 8 | 0.0309 |  |  |
|  | T | 6.1921 | 1 | 6.1921 | 217.3025 | 0.0000 |
|  | FS x T | 0.2707 | 1 | 0.2707 | 9.4996 | 0.0095 |
|  | G x T | 1.7266 | 2 | 0.8633 | 30.2964 | 0.0000 |
|  | FS x G x T | 0.3873 | 2 | 0.1937 | 6.7960 | 0.0106 |
|  | Error III | 0.3419 | 12 | 0.0285 |  |  |
|  | FS | 98.9471 | 1 | 98.9471 | 79.0293 | 0.0009 |
| 2,5-hydroxybenzoic acid | Error I | 5.0081 | 4 | 1.2520 |  |  |
|  | G | 1348.4453 | 2 | 674.2227 | 36.5299 | 0.0001 |
|  | FS x G | 23.6217 | 2 | 11.8109 | 0.6399 | 0.5523 |
|  | Error II | 147.6539 | 8 | 18.4567 |  |  |
|  | T | 98.8933 | 1 | 98.8933 | 12.0727 | 0.0046 |
|  | FS x T | 21.7472 | 1 | 21.7472 | 2.6549 | 0.1292 |
|  | G x T | 165.7994 | 2 | 82.8997 | 10.1202 | 0.0027 |
|  | FS x G x T | 14.1677 | 2 | 7.0839 | 0.8648 | 0.4458 |
|  | Error III | 98.2978 | 12 | 8.1915 |  |  |
| 4-hydroxybenzoic acid | FS | 11.8108 | 1 | 11.8108 | 3.9307 | 0.1184 |
|  | Error I | 12.0190 | 4 | 3.0048 |  |  |
|  | G | 5.7082 | 2 | 2.8541 | 1.8780 | 0.2144 |
|  | FS x G | 2.5708 | 2 | 1.2854 | 0.8458 | 0.4643 |
|  | Error II | 12.1580 | 8 | 1.5198 |  |  |
|  | T | 21.9107 | 1 | 21.9107 | 19.7557 | 0.0008 |
|  | FS x T | 7.8369 | 1 | 7.8369 | 7.0661 | 0.0209 |
|  | G x T | 20.8317 | 2 | 10.4159 | 9.3914 | 0.0035 |
|  | FS x G x T | 3.8508 | 2 | 1.9254 | 1.7360 | 0.2177 |
|  | Error III | 13.3090 | 12 | 1.1091 |  |  |
| Caffeic acid | FS | 0.0898 | 1 | 0.0898 | 26.3894 | 0.0068 |
|  | Error I | 0.0136 | 4 | 0.0034 |  |  |
|  | G | 0.0247 | 2 | 0.0123 | 6.6837 | 0.0196 |
|  | FS x G | 0.0003 | 2 | 0.0001 | 0.0724 | 0.9308 |
|  | Error II | 0.0148 | 8 | 0.0018 |  |  |
|  | T | 3.4065 | 1 | 3.4065 | 1453.5199 | 0.0000 |
|  | FS x T | 0.0110 | 1 | 0.0110 | 4.6978 | 0.0510 |
|  | G x T | 1.8135 | 2 | 0.9068 | 386.9067 | 0.0000 |
|  | FS x G x T | 0.0285 | 2 | 0.0142 | 6.0706 | 0.0151 |
|  | Error III | 0.0281 | 12 | 0.0023 |  |  |
| Syringic acid | FS | 43.1822 | 1 | 43.1822 | 6.9253 | 0.0581 |
|  | Error I | 24.9418 | 4 | 6.2354 |  |  |
|  | G | 4610.7433 | 2 | 2305.3716 | 473.7749 | 0.0000 |
|  | FS x G | 7.1765 | 2 | 3.5883 | 0.7374 | 0.5082 |
|  | Error II | 38.9277 | 8 | 4.8660 |  |  |
|  | T | 8.0465 | 1 | 8.0465 | 1.7252 | 0.2136 |
|  | FS x T | 0.0102 | 1 | 0.0102 | 0.0022 | 0.9634 |
|  | G x T | 12.1964 | 2 | 6.0982 | 1.3075 | 0.3064 |
|  | FS x G x T | 7.8149 | 2 | 3.9074 | 0.8378 | 0.4565 |
|  | Error III | 55.9693 | 12 | 4.6641 |  |  |
| p-Coumaric acid | FS | 0.0023 | 1 | 0.0023 | 82.0899 | 0.0008 |
|  | Error I | 0.0001 | 4 | 0.0000 |  |  |
|  | G | 0.0015 | 2 | 0.0008 | 3.2714 | 0.0916 |
|  | FS x G | 0.0005 | 2 | 0.0003 | 1.1030 | 0.3775 |
|  | Error II | 0.0019 | 8 | 0.0002 |  |  |
|  | T | 0.0004 | 1 | 0.0004 | 2.6652 | 0.1285 |
|  | FS x T | 0.0006 | 1 | 0.0006 | 4.2686 | 0.0611 |
|  | G x T | 0.0001 | 2 | 0.0000 | 0.2011 | 0.8206 |
|  | FS x G x T | 0.0004 | 2 | 0.0002 | 1.2464 | 0.3222 |
|  | Error III | 0.0017 | 12 | 0.0001 |  |  |
| Chlorogenic acid | FS | 0.0374 | 1 | 0.0374 | 16.8851 | 0.0147 |
|  | Error I | 0.0089 | 4 | 0.0022 |  |  |
|  | G | 0.0441 | 2 | 0.0220 | 16.5142 | 0.0014 |
|  | FS x G | 0.0453 | 2 | 0.0227 | 16.9831 | 0.0013 |
|  | Error II | 0.0107 | 8 | 0.0013 |  |  |
|  | T | 0.0485 | 1 | 0.0485 | 38.4889 | 0.0000 |
|  | FS x T | 0.0107 | 1 | 0.0107 | 8.4546 | 0.0131 |
|  | G x T | 0.0284 | 2 | 0.0142 | 11.2752 | 0.0018 |
|  | FS x G x T | 0.0234 | 2 | 0.0117 | 9.2753 | 0.0037 |
|  | Error III | 0.0151 | 12 | 0.0013 |  |  |
| Protocatechuic acid | FS | 16.9051 | 1 | 16.9051 | 261.0080 | 0.0001 |
|  | Error I | 0.2591 | 4 | 0.0648 |  |  |
|  | G | 41.6386 | 2 | 20.8193 | 412.6261 | 0.0000 |
|  | FS x G | 2.3460 | 2 | 1.1730 | 23.2485 | 0.0005 |
|  | Error II | 0.4036 | 8 | 0.0505 |  |  |
|  | T | 0.7514 | 1 | 0.7514 | 11.0362 | 0.0061 |
|  | FS x T | 0.9814 | 1 | 0.9814 | 14.4138 | 0.0025 |
|  | G x T | 1.5523 | 2 | 0.7762 | 11.3999 | 0.0017 |
|  | FS x G x T | 2.7802 | 2 | 1.3901 | 20.4169 | 0.0001 |
|  | Error III | 0.8170 | 12 | 0.0681 |  |  |
| Synaptic acid | FS | 3.6240 | 1 | 3.6240 | 79.9725 | 0.0009 |
|  | Error I | 0.1813 | 4 | 0.0453 |  |  |
|  | G | 17.5474 | 2 | 8.7737 | 68.7527 | 0.0000 |
|  | FS x G | 1.5179 | 2 | 0.7589 | 5.9473 | 0.0261 |
|  | Error II | 1.0209 | 8 | 0.1276 |  |  |
|  | T | 0.0027 | 1 | 0.0027 | 0.0100 | 0.9219 |
|  | FS x T | 5.0654 | 1 | 5.0654 | 18.7874 | 0.0010 |
|  | G x T | 15.4477 | 2 | 7.7238 | 28.6474 | 0.0000 |
|  | FS x G x T | 12.4802 | 2 | 6.2401 | 23.1443 | 0.0001 |
|  | Error III | 3.2354 | 12 | 0.2696 |  |  |
| Ferulic acid | FS | 993160.8339 | 1 | 993160.8339 | 540.7205 | 0.0000 |
|  | Error I | 7346.9446 | 4 | 1836.7362 |  |  |
|  | G | 1862620.3986 | 2 | 931310.1993 | 641.0880 | 0.0000 |
|  | FS x G | 164980.6996 | 2 | 82490.3498 | 56.7841 | 0.0000 |
|  | Error II | 11621.6210 | 8 | 1452.7026 |  |  |
|  | T | 670933.5412 | 1 | 670933.5412 | 226.9765 | 0.0000 |
|  | FS x T | 31479.7476 | 1 | 31479.7476 | 10.6496 | 0.0068 |
|  | G x T | 568457.7737 | 2 | 284228.8869 | 96.1545 | 0.0000 |
|  | FS x G x T | 43040.3687 | 2 | 21520.1844 | 7.2803 | 0.0085 |
|  | Error III | 35471.5202 | 12 | 2955.9600 |  |  |
| Naringenin | FS | 446.9807 | 1 | 446.9807 | 50.1054 | 0.0021 |
|  | Error I | 35.6832 | 4 | 8.9208 |  |  |
|  | G | 17896.3867 | 2 | 8948.1933 | 1569.5121 | 0.0000 |
|  | FS x G | 4.2864 | 2 | 2.1432 | 0.3759 | 0.6982 |
|  | Error II | 45.6101 | 8 | 5.7013 |  |  |
|  | T | 494.4089 | 1 | 494.4089 | 53.8119 | 0.0000 |
|  | FS x T | 3.4115 | 1 | 3.4115 | 0.3713 | 0.5537 |
|  | G x T | 221.9666 | 2 | 110.9833 | 12.0795 | 0.0013 |
|  | FS x G x T | 13.9338 | 2 | 6.9669 | 0.7583 | 0.4897 |
|  | Error III | 110.2528 | 12 | 9.1877 |  |  |
| Vitaxin | FS | 14.2830 | 1 | 14.2830 | 1459.4114 | 0.0000 |
|  | Error I | 0.0391 | 4 | 0.0098 |  |  |
|  | G | 9.0978 | 2 | 4.5489 | 181.7965 | 0.0000 |
|  | FS x G | 2.5569 | 2 | 1.2784 | 51.0920 | 0.0000 |
|  | Error II | 0.2002 | 8 | 0.0250 |  |  |
|  | T | 0.2716 | 1 | 0.2716 | 11.3027 | 0.0057 |
|  | FS x T | 0.0161 | 1 | 0.0161 | 0.6698 | 0.4291 |
|  | G x T | 1.0973 | 2 | 0.5486 | 22.8333 | 0.0001 |
|  | FS x G x T | 0.4774 | 2 | 0.2387 | 9.9340 | 0.0029 |
|  | Error III | 0.2883 | 12 | 0.0240 |  |  |
| Rutin | FS | 8.9048 | 1 | 8.9048 | 2.1930 | 0.2128 |
|  | Error I | 16.2423 | 4 | 4.0606 |  |  |
|  | G | 38.0092 | 2 | 19.0046 | 1.8128 | 0.2242 |
|  | FS x G | 6.4721 | 2 | 3.2361 | 0.3087 | 0.7428 |
|  | Error II | 83.8683 | 8 | 10.4835 |  |  |
|  | T | 15.3641 | 1 | 15.3641 | 2.0362 | 0.1791 |
|  | FS x T | 4.6798 | 1 | 4.6798 | 0.6202 | 0.4462 |
|  | G x T | 12.9549 | 2 | 6.4774 | 0.8585 | 0.4483 |
|  | FS x G x T | 4.5616 | 2 | 2.2808 | 0.3023 | 0.7446 |
|  | Error III | 90.5447 | 12 | 7.5454 |  |  |
| Quercetin | FS | 481.5461 | 1 | 481.5461 | 34.7292 | 0.0041 |
|  | Error I | 55.4630 | 4 | 13.8658 |  |  |
|  | G | 9347.8929 | 2 | 4673.9465 | 429.3479 | 0.0000 |
|  | FS x G | 162.7502 | 2 | 81.3751 | 7.4751 | 0.0148 |
|  | Error II | 87.0892 | 8 | 10.8862 |  |  |
|  | T | 1.3979 | 1 | 1.3979 | 0.0834 | 0.7777 |
|  | FS x T | 119.3879 | 1 | 119.3879 | 7.1205 | 0.0205 |
|  | G x T | 152.5941 | 2 | 76.2970 | 4.5505 | 0.0338 |
|  | FS x G x T | 44.6967 | 2 | 22.3484 | 1.3329 | 0.3001 |
|  | Error III | 201.2014 | 12 | 16.7668 |  |  |
| Apigenin | FS | 3419.1174 | 1 | 3419.1174 | 112.3643 | 0.0004 |
|  | Error I | 121.7155 | 4 | 30.4289 |  |  |
|  | G | 2329.8608 | 2 | 1164.9304 | 43.3447 | 0.0001 |
|  | FS x G | 1771.9761 | 2 | 885.9881 | 32.9658 | 0.0001 |
|  | Error II | 215.0079 | 8 | 26.8760 |  |  |
|  | T | 129.9254 | 1 | 129.9254 | 4.9197 | 0.0466 |
|  | FS x T | 588.6805 | 1 | 588.6805 | 22.2907 | 0.0005 |
|  | G x T | 17602.1910 | 2 | 8801.0955 | 333.2574 | 0.0000 |
|  | FS x G x T | 595.1256 | 2 | 297.5628 | 11.2673 | 0.0018 |
|  | Error III | 316.9116 | 12 | 26.4093 |  |  |
| Campferol | FS | 87.6720 | 1 | 87.6720 | 259.2413 | 0.0001 |
|  | Error I | 1.3527 | 4 | 0.3382 |  |  |
|  | G | 15.7971 | 2 | 7.8986 | 50.5551 | 0.0000 |
|  | FS x G | 39.6394 | 2 | 19.8197 | 126.8569 | 0.0000 |
|  | Error II | 1.2499 | 8 | 0.1562 |  |  |
|  | T | 8.6792 | 1 | 8.6792 | 45.3721 | 0.0000 |
|  | FS x T | 0.3853 | 1 | 0.3853 | 2.0142 | 0.1813 |
|  | G x T | 0.5664 | 2 | 0.2832 | 1.4805 | 0.2663 |
|  | FS x G x T | 15.4868 | 2 | 7.7434 | 40.4797 | 0.0000 |
|  | Error III | 2.2955 | 12 | 0.1913 |  |  |
| Luteolin | FS | 75.5645 | 1 | 75.5645 | 1096.2404 | 0.0000 |
|  | Error I | 0.2757 | 4 | 0.0689 |  |  |
|  | G | 394.1819 | 2 | 197.0910 | 764.9063 | 0.0000 |
|  | FS x G | 9.4116 | 2 | 4.7058 | 18.2632 | 0.0010 |
|  | Error II | 2.0613 | 8 | 0.2577 |  |  |
|  | T | 3.5663 | 1 | 3.5663 | 29.6651 | 0.0001 |
|  | FS x T | 1.2924 | 1 | 1.2924 | 10.7504 | 0.0066 |
|  | G x T | 3.4100 | 2 | 1.7050 | 14.1824 | 0.0007 |
|  | FS x G x T | 1.0020 | 2 | 0.5010 | 4.1675 | 0.0422 |
|  | Error III | 1.4426 | 12 | 0.1202 |  |  |
| Chlorophull *a* | FS | 0.0150 | 1 | 0.0150 | 40.6976 | 0.0031 |
|  | Error I | 0.0015 | 4 | 0.0004 |  |  |
|  | G | 3.7212 | 2 | 1.8606 | 5667.1540 | 0.0000 |
|  | FS x G | 0.0100 | 2 | 0.0050 | 15.1681 | 0.0019 |
|  | Error II | 0.0026 | 8 | 0.0003 |  |  |
|  | T | 1.0183 | 1 | 1.0183 | 865.7900 | 0.0000 |
|  | FS x T | 0.0095 | 1 | 0.0095 | 8.0599 | 0.0149 |
|  | G x T | 0.5268 | 2 | 0.2634 | 223.9310 | 0.0000 |
|  | FS x G x T | 0.0153 | 2 | 0.0077 | 6.5217 | 0.0121 |
|  | Error III | 0.0141 | 12 | 0.0012 |  |  |
| Chlorophull *b* | FS | 0.0008 | 1 | 0.0008 | 185.1353 | 0.0002 |
|  | Error I | 0.0000 | 4 | 0.0000 |  |  |
|  | G | 0.0103 | 2 | 0.0051 | 214.6820 | 0.0000 |
|  | FS x G | 0.0004 | 2 | 0.0002 | 8.9077 | 0.0092 |
|  | Error II | 0.0002 | 8 | 0.0000 |  |  |
|  | T | 0.0203 | 1 | 0.0203 | 1166.4809 | 0.0000 |
|  | FS x T | 0.0008 | 1 | 0.0008 | 46.0959 | 0.0000 |
|  | G x T | 0.0103 | 2 | 0.0051 | 295.2968 | 0.0000 |
|  | FS x G x T | 0.0004 | 2 | 0.0002 | 12.2526 | 0.0013 |
|  | Error III | 0.0002 | 12 | 0.0000 |  |  |
| Delphinidin | FS | 0.3428 | 1 | 0.3428 | 28.3780 | 0.0060 |
|  | Error I | 0.0483 | 4 | 0.0121 |  |  |
|  | G | 16.1351 | 2 | 8.0676 | 543.7459 | 0.0000 |
|  | FS x G | 0.2922 | 2 | 0.1461 | 9.8486 | 0.0070 |
|  | Error II | 0.1187 | 8 | 0.0148 |  |  |
|  | T | 0.3661 | 1 | 0.3661 | 297.6908 | 0.0000 |
|  | FS x T | 0.0070 | 1 | 0.0070 | 5.6596 | 0.0348 |
|  | G x T | 0.9385 | 2 | 0.4693 | 381.6052 | 0.0000 |
|  | FS x G x T | 0.0046 | 2 | 0.0023 | 1.8670 | 0.1968 |
|  | Error III | 0.0148 | 12 | 0.0012 |  |  |
| Luteolinidine | FS | 0.0041 | 1 | 0.0041 | 13.4554 | 0.0214 |
|  | Error I | 0.0012 | 4 | 0.0003 |  |  |
|  | G | 0.3641 | 2 | 0.1820 | 702.8697 | 0.0000 |
|  | FS x G | 0.0061 | 2 | 0.0031 | 11.8279 | 0.0041 |
|  | Error II | 0.0021 | 8 | 0.0003 |  |  |
|  | T | 0.0151 | 1 | 0.0151 | 44.3073 | 0.0000 |
|  | FS x T | 0.0005 | 1 | 0.0005 | 1.6157 | 0.2278 |
|  | G x T | 0.0350 | 2 | 0.0175 | 51.4511 | 0.0000 |
|  | FS x G x T | 0.0014 | 2 | 0.0007 | 2.0580 | 0.1704 |
|  | Error III | 0.0041 | 12 | 0.0003 |  |  |
| Phytomelanin | FS | 1.0612 | 1 | 1.0612 | 35.6448 | 0.0040 |
|  | Error I | 0.1191 | 4 | 0.0298 |  |  |
|  | G | 447.3144 | 2 | 223.6572 | 14780.0737 | 0.0000 |
|  | FS x G | 0.0929 | 2 | 0.0464 | 3.0696 | 0.1025 |
|  | Error II | 0.1211 | 8 | 0.0151 |  |  |
|  | T | 59.5095 | 1 | 59.5095 | 1650.1499 | 0.0000 |
|  | FS x T | 1.0481 | 1 | 1.0481 | 29.0623 | 0.0002 |
|  | G x T | 21.1656 | 2 | 10.5828 | 293.4524 | 0.0000 |
|  | FS x G x T | 0.4032 | 2 | 0.2016 | 5.5896 | 0.0193 |
|  | Error III | 0.4328 | 12 | 0.0361 |  |  |
| ABTS^+^ | FS | 0.0032 | 1 | 0.0032 | 0.4877 | 0.5234 |
|  | Error I | 0.0263 | 4 | 0.0066 |  |  |
|  | G | 0.5834 | 2 | 0.2917 | 49.7189 | 0.0000 |
|  | FS x G | 0.0198 | 2 | 0.0099 | 1.6833 | 0.2454 |
|  | Error II | 0.0469 | 8 | 0.0059 |  |  |
|  | T | 0.0621 | 1 | 0.0621 | 7.6279 | 0.0172 |
|  | FS x T | 0.0003 | 1 | 0.0003 | 0.0427 | 0.8398 |
|  | G x T | 0.0924 | 2 | 0.0462 | 5.6730 | 0.0184 |
|  | FS x G x T | 0.0144 | 2 | 0.0072 | 0.8855 | 0.4378 |
|  | Error III | 0.0978 | 12 | 0.0081 |  |  |
